# Impact of caregiver and parenting status on time trade-off and standard gamble utility scores for health state descriptions

**DOI:** 10.1186/1477-7525-12-48

**Published:** 2014-04-09

**Authors:** Louis S Matza, Kristina S Boye, David H Feeny, Joseph A Johnston, Lee Bowman, Jessica B Jordan

**Affiliations:** 1 Senior Research Scientist, Outcomes Research, Evidera, Bethesda, MD, USA; 2 Sr. Research Advisor, Global Health Outcomes, Diabetes, Eli Lilly and Company, Indianapolis, IN, USA; 3 Department of Economics (Professor Emeritus), University of Alberta, Edmonton, AB, Canada; 4 Medical Fellow, Global Health Outcomes, Eli Lilly and Company, Indianapolis, IN, USA; 5 Research Fellow, Global Health Outcomes, Oncology, Eli Lilly and Company, Indianapolis, IN, USA; 6 Senior Research Associate, Outcomes Research, Evidera, Bethesda, MD, USA

**Keywords:** Utility, Time trade-off, Standard gamble, Caregiver, Parenting, Cost-utility

## Abstract

**Background:**

The purpose of this study was to examine the effect of caregiver status on time trade-off (TTO) and standard gamble (SG) health state utility scores. Respondents were categorized as caregivers if they reported that either children or adults depended on them for care.

**Methods:**

This study was a secondary analysis of data from three studies in which general population samples rated health state descriptions. Study 1: UK; four osteoarthritis health states. Study 2: UK; three adult ADHD health states. Study 3: US; 16 schizophrenia health states. All three studies included time trade-off assessment. Study 1 also included standard gamble. Descriptive statistics were calculated to examine willingness to trade in TTO or gamble in SG. Utilities for caregivers and non-caregivers were compared using t-tests and ANCOVA models.

**Results:**

There were 364 respondents including 106 caregivers (n = 30, 47, and 29 in Studies 1, 2, and 3) and 258 non-caregivers. Most caregivers were parents of dependent children (78.3%). Compared to non-caregivers, caregivers had more responses at the ceiling (i.e., utility = 0.95), indicating less willingness to trade time or gamble. All utilities were higher for caregivers than non-caregivers (mean utility difference between groups: 0.07 to 0.16 in Study 1 TTO; 0.03 to 0.17 in Study 1 SG; 0.06 to 0.10 in Study 2 TTO; 0.11 to 0.22 in Study 3 TTO). These differences were statistically significant for at least two health states in each study (p < 0.05). Results of sensitivity analyses with two caregiver subgroups (parents of dependent children and parents of any child regardless of whether the child was still dependent) followed the same pattern as results of the primary analysis. The parent subgroups were generally less willing to trade time or gamble (i.e., resulting in higher utility scores) than comparison groups of non-parents.

**Conclusions:**

Results indicate that caregiver status, including being a parent, influences responses in time trade-off health state valuation. Caregivers (i.e., predominantly parents) were less willing than non-caregivers to trade time, resulting in higher utility scores. This pattern was consistent across multiple health states in three studies. Standard gamble results followed similar patterns, but with less consistent differences between groups. It may be useful to consider parenting/caregiving status when collecting, interpreting, or using utility data because this demographic variable could influence results.

## Background

A cost-utility model is a type of cost-effectiveness analysis that incorporates the preferences of individuals for various health states, and these preferences are quantified in terms of utilities [1-3]. Healthcare policy and reimbursement decisions are often directly informed by results of cost-utility models, and the results of these models depend on the utility scores. Therefore, it is important to understand factors that may influence respondents’ decisions in tasks designed to elicit utilities.

There is a substantial body of research on respondent characteristics that may influence health state preferences and utility scores. Characteristics that have been found in at least one study to influence utilities include experience of the health state being rated [4,5], education level [6,7], religious beliefs [8-10], age [7,11,12], gender [11], marital status [11], race [13], and culture [14].

One participant characteristic that has received relatively little attention is whether the respondent is the caregiver of other individuals such as young children. One previous study examined the extent to which being a mother of a young child influenced responses to time trade-off (TTO) utility assessment [15]. A group of 30 mothers was compared to a large general population sample, and results indicated that the mothers were not willing to trade as much time as the general population sample. Thus, the small sample of mothers had significantly higher mean utilities for four generic EQ-5D health states than the general population. The authors interpreted this difference as suggesting that parenting could affect one’s extrinsic goals, which could influence preferences during health state valuation.

The purpose of the current study was to analyze data from three utility studies to examine further the influence of caregiver status, including being a parent, on utilities. Because these three utility studies were conducted with different health states, the current analysis can provide insight into whether the impact of caregiver status is consistent across multiple samples and health states representing a range of health conditions. It was expected that caregivers, compared to non-caregivers, would be more resistant to trading time in TTO utility assessment and more resistant to gambling in standard gamble utility assessment. These tendencies were expected to result in higher utilities for the same health states for caregivers than for non-caregivers.

## Methods

### Overview of study design

The current study is a secondary analysis of data from three studies in which general population respondents completed utility interviews to rate a series of health state descriptions. In Study 1, participants in London rated four osteoarthritis health states in time trade-off and standard gamble (SG) tasks. These health states varied by severity of osteoarthritis symptoms. In Study 2, participants in London and Edinburgh rated three adult attention-deficit/hyperactivity disorder (ADHD) health states in TTO interviews. These health states described treatment response, treatment non-response, and untreated adult ADHD. In Study 3, participants in San Francisco rated 16 health states in TTO interviews. These health states described schizophrenia and antipsychotic treatment with a range of adverse events. All TTO utility assessment procedures were consistent across the three studies, including the use of a 10-year time horizon. Table 1 provides an overview of the three studies used in this secondary analysis.

**Table 1:** Data sources for the current secondary analysis

**Study characteristics**	**Study 1**	**Study 2**	**Study 3**
**Number of health states**	4	3	16
**Description of health states**	Osteoarthritis: three severity levels, plus one adverse event	Adult ADHD: responder, non-responder, untreated	Schizophrenia with a range of treatment-related adverse events
**Utility assessment method**	TTO, SG	TTO	TTO
**Geographic location**	London (UK)	London and Edinburgh (UK)	San Francisco (US)
**Dates of data collection**	November 2011	October 2012	April-May 2012
**Sample size**			
Total	80	158	126
Caregivers	30	47	29
Non-caregivers	50	111	97

TTO = Time trade-off; SG = Standard gamble; UK = United Kingdom; US = United States; ADHD = Attention deficit/hyperactivity disorder.

For the current analyses, participants were categorized as caregivers or non-caregivers. Respondents were asked “Do you have any children?” If they responded “yes,” they were asked “How many of these children still depend on you to care for them?” Finally, they were asked “Are there any other people besides children who depend on you to care for them (for example, elderly or disabled relatives)?” Participants were considered to be caregivers if they reported that at least one child or one adult depended on them for care. Otherwise, they were classified as non-caregivers.

### Participants

In all three studies, participants were required to be (1) at least 18 years old; (2) able to understand the assessment procedures; and (3) able and willing to give written informed consent. Participants were not eligible if they had cognitive impairment, hearing difficulty, visual impairment, severe psychopathology, or insufficient knowledge of English that could interfere with the ability to complete study measures. Inclusion criteria did not specify any particular clinical characteristics, which is consistent with recommendations from agencies like the UK’s National Institute for Health and Care Excellence (NICE), most of which prefer that utilities represent general population preferences [16-18]. Participants were recruited through newspaper and online classified advertisements.

#### Study 1

A total of 197 potential participants responded to advertisements, and 109 of these were reached for screening. Of the 109 screened participants, two were ineligible, 101 were scheduled for interviews, and 81 attended interviews in London, UK. Of the 81 participants, one was unable to complete the utility interview procedures. Therefore, the Study 1 analysis was conducted with the 80 participants who completed the interview (i.e., 40.6% of individuals who initially responded to the advertisements).

#### Study 2

A total of 396 potential participants responded to advertisements, and 203 of these were reached for screening. Of the 203 screened participants, 199 were eligible, 174 were scheduled (88 in Edinburgh and 86 in London) for interviews, and 160 participants attended interviews (81 in Edinburgh and 79 in London). Two of the 160 participants were unable to complete the utility interview. Thus, a total of 158 completed utility interviews were used in the analyses (i.e., 39.9% of individuals who initially responded to the advertisements), including 80 in Edinburgh and 78 in London.

#### Study 3

A total of 376 participants responded to the advertisements, and 202 of these were reached for screening. Of the 202 screened participants, 192 were eligible, 136 were scheduled for interviews, and 128 participants attended interviews in San Francisco, US. Two of the 128 participants were unable to complete the utility interview. Therefore, the Study 3 analysis was based on the 126 participants who completed the interview (i.e., 33.5% of individuals who initially responded to the advertisements).

### Health states

#### Study 1

Four osteoarthritis health states associated with elective total hip arthroplasty were presented during the utility interview in Study 1. The first three health states described patients with mild (health state A), moderate (B), or severe (C) osteoarthritis of the hip. These health states were based on health states presented in two previous studies [19,20], with minor edits made so that they would be appropriate for administration in the UK. A fourth health state (D) was identical to health state B, except for the addition of an adverse event: “Because of your medication, you occasionally have an upset stomach that causes discomfort and loss of appetite.”

#### Study 2

Three adult ADHD health states were presented during the utility interview in Study 2: (A) Adult ADHD, receiving medication treatment, responder; (B): Adult ADHD, receiving medication treatment, non-responder; and (C): Adult ADHD, untreated. These health states were drafted based on literature review, four clinician interviews (all four with MD degrees; two of whom also had PhD degrees), and pooled data from six clinical trials. The literature review was conducted first to ensure that the health states would be grounded in clinical evidence. This literature was then used to draft structured interview guides for the clinician interviews. The data from the clinical trials was examined to ensure that health states accurately described differences between treatment responders and non-responders.

#### Study 3

In order to estimate the disutility associated with adverse events commonly associated with antipsychotic treatment for schizophrenia, participants in Study 3 first rated health state A (i.e., the “basic health state”), which described stable schizophrenia without an adverse event. Then, 15 additional health states were rated, each including the basic health state plus one treatment-related adverse event. These health states were drafted based on literature review and a series of interviews with three clinicians (two with MD degrees and one with a PharmD) who had experience treating patients with schizophrenia.

### Utility interview procedures and scoring

Interviews were conducted in London for Study 1, in London and Edinburgh for Study 2, and in San Francisco for Study 3 (see Table 1 for dates of data collection). All procedures were approved by an independent Institutional Review Board, and participants provided written informed consent prior to completing any part of each study.

First, participants in each study rated the health states in a visual analogue scale (VAS) or ranking exercise that was intended to introduce participants to each of the health state descriptions. In the VAS and ranking exercises, health states were presented to respondents in random order. Then, health state utilities were obtained using TTO methods for all three studies, in addition to SG methods for Study 1. In Study 1, half of the participants were randomly assigned to complete the TTO task first, while the other half completed the SG task first. In TTO and SG procedures, health states were presented in the order in which they were previously ranked by participants, proceeding from best to worst.

TTO and SG interviews followed generally accepted procedures, which have previously been described in detail [1,21]. In the TTO task, participants were offered a choice between spending 10 years in the health state being rated versus spending varying shorter amounts of time in full health, followed by death. Time was varied in 1-year increments. The utility score was calculated based on the choice in which the respondent was indifferent between *y* years in the health state being evaluated (i.e., 10 years) and *x* years in full health (followed by dead). The resulting utility estimate (*u*) is calculated as *u* = *x*/*y*.

In the SG task in Study 1, participants were offered a choice between two alternatives, one that was certain and one that was uncertain. Choice A was to remain in the health state being rated for 10 years. Choice B was the uncertain choice with two possible outcomes; either to (1) remain in full health for the 10-year period with a probability of *p* or (2) death with a probability of 1 – *p*. Probability *p* was varied in 10% increments until the participant was indifferent between choices A and B, and the resulting utility score was calculated based on *p* at this point of indifference.

In Study 1, precise utility scores were not obtained for health states considered worse than being dead for each respondent (i.e., negative utility scores). Therefore, “worse than dead” ratings in Study 1 are used in categorical analyses (e.g., counted among the frequencies of utilities less than 0.95), but not in continuous analyses which require precise utility scores.

In Studies 2 and 3, if participants indicated that a health state was worse than being dead, the interviewer altered the task so that respondents were offered a choice between immediate death (alternative 1) and a 10-year life span (alternative 2) beginning with varying amounts of time in the health state being rated, followed by full health for the remainder of the 10-year timeframe. For these health states that received negative utility scores, the current study used a bounded scoring approach, which is commonly used [21]. This scoring approach limits the score range of negative scores between 0 and −1. To compute these utilities, analyses of Studies 2 and 3 used the Dolan [22] method as described by Rowen & Brazier [1]. This method uses the formula *u* = −*x*/*t*, where *x* is the number of years in full health, and *t* is the total life span of alternative 2 in the TTO choice. In the current study, *t* was 10 years, which is equal to the number of years in the health state being rated plus subsequent years in full health.

### Statistical analysis procedures

Statistical analyses were completed using SAS version 8.12 (SAS Institute, Cary, NC). For each of the three studies, demographic variables, willingness to trade (in TTO), willingness to gamble (in SG for Study 1 only), and mean utilities are reported for two subgroups (caregivers and non-caregivers, categorized as described in the section above titled “Overview of Study Design”). Caregivers and non-caregivers were compared in terms of willingness to trade in TTO and willingness to gamble in SG with chi-square analyses comparing rates of utility scores below 0.95. A score of 0.95 indicates that a respondent was unwilling to trade or gamble, while a score below 0.95 indicates that a respondent was willing to trade or gamble. Utilities for caregivers and non-caregivers were compared using t-tests. After completing the initial t-tests, the utility comparisons were re-run as analyses of covariance (ANCOVA models) controlling for demographic variables that were significantly different between the two subgroups (i.e., gender and marital status in Studies 1 and 2; ethnicity and marital status in Study 3).

Sensitivity analyses were conducted in order to ascertain whether results for parents were similar to results for caregivers in general. All analyses described above were re-run twice, with two different ways of categorizing the sample. First, analyses were conducted to examine only the caregivers who were parents of dependent children, rather than the larger group of caregivers of adults and/or children that was the focus of the main analysis described above. These parents of dependent children were compared to all other respondents. Second, analyses were re-run focusing on all parents in the sample, regardless of whether their children still depended on them for care. These parents were compared to all non-parents in the sample.

## Results

### Sample description

In total, the current analysis was conducted with data from 364 general population respondents across the three studies, including 106 (29.1%) categorized as caregivers and 258 (70.9%) categorized as non-caregivers. Of the 30 participants with dependents in Study 1, 25 reported that they were parents of dependent children, while the other five reported being caregivers of adults. In Study 1, there were no statistically significant differences between caregivers (n = 30) and non-caregivers (n = 50) in age, ethnicity, employment status, or education level (Table 2). However, there were significant between-group differences in gender and marital status. Compared to non-caregivers, the caregiver group was more likely to be male (p = 0.03) and less likely to be single (p = 0.02).

**Table 2:** Demographic characteristics

**Characteristics**	**Study 1**			**Study 2**			**Study 3**		
	**Caregivers (N = 30)**	**Non-caregivers (N = 50)**	**p-value**^**‡**^	**Caregivers (N = 47)**	**Non-caregivers (N = 111)**	**p-value**^**‡**^	**Caregivers (N = 29)**	**Non-caregivers (N = 97)**	**p-value**^**‡**^
**Age (mean, SD)**	48.3 (11.5)	46.7 (15.9)	0.64	44.9 (11.1)	47.9 (15.6)	0.23	48.9 (11.2)	49.7 (16.9)	0.82
**Gender (n,%)**									
Male	20 (66.7%)	21 (42.0%)	0.03	15 (31.9%)	65 (58.6%)	<0.01	12 (41.4%)	48 (49.5%)	0.44
Female	10 (33.3%)	29 (58.0%)		32 (68.1%)	46 (41.4%)		17 (58.6%)	49 (50.5%)	
Ethnicity (n,%)^*^									
White	15 (50.0%)	35 (70.0%)	0.07	41 (87.2%)	91 (82.0%)	0.42	10 (34.5%)	58 (59.8%)	0.02
Other	15 (50.0%)	15 (30.0%)		6 (12.8%)	20 (18.0%)		19 (65.5%)	39 (40.2%)	
**Marital status (n,%)**									
Single	8 (26.7%)	29 (58.0%)	0.02	10 (21.3%)	54 (48.6%)	<0.01	9 (31.0%)	59 (60.8%)	0.02
Married/Living with partner	11 (36.7%)	12 (24.0%)		26 (55.3%)	35 (31.5%)		8 (27.6%)	13 (13.4%)	
Divorced/Separated/Widowed	11 (36.7%)	9 (18.0%)		11 (23.4%)	22 (19.8%)		12 (41.4%)	25 (25.8%)	
**Employment status (n,%)**^**†**^									
Full-time work	8 (26.7%)	12 (24.0%)	0.12	17 (36.2%)	35 (31.5%)	0.11	7 (24.1%)	16 (16.5%)	0.15
Part-time work	10 (33.3%)	18 (36.0%)		16 (34.0%)	24 (21.6%)		12 (41.4%)	24 (24.7%)	
Unemployed	1 (3.3%)	10 (20.0%)		2 (4.3%)	17 (15.3%)		3 (10.3%)	17 (17.5%)	
Other	11 (36.7%)	10 (20.0%)		12 (25.5%)	35 (31.5%)		7 (24.1%)	40 (41.2%)	
**Education Level (n,%)**									
No university degree	16 (53.3%)	25 (50.0%)	0.77	34 (72.3%)	64 (57.7%)	0.08	14 (48.3%)	44 (45.4%)	0.78
University or postgraduate degree	14 (46.7%)	25 (50.0%)		13 (27.7%)	47 (42.3%)		15 (51.7%)	53 (54.6%)	

^*^For Study 1, other ethnicity includes 13 Black, 8 Asian, and 9 other races. For Study 2, other ethnicity includes 7 Black, 8 Asian, 1 Chinese and 10 mixed races. For Study 3, other ethnicity includes 24 Black, 10 Hispanic/Latino, 10 Asian, 5 Hawaiian/Pacific Islander and 9 other races.

^†^For Study 1, other employment includes 3 homemakers, 2 students, 15 retired and 1 disabled. For Study 2, other employment includes 8 homemakers, 9 students, 27 retired, and 3 disabled. For Study 3, other employment includes 3 homemakers, 8 students, 15 retired, 19 disabled and 1 other.

^‡^P-values are for the comparison between caregivers and non-caregivers. Continuous variables were compared with t-tests, and categorical variables were compared with chi-square analysis.

In Study 2, 47 of the 158 participants were categorized as caregivers. Of the 47, 39 reported that they were parents of dependent children, four were caregivers of adults, and another four reported that they were both parents of dependent children and caregivers of adults (i.e., a total of 43 respondents were parents of dependent children). Similar to Study 1, there were no statistically significant between-group differences in age, ethnicity, employment status, or education level (Table 2). However, there were significant differences in gender and marital status. Compared to the group without dependents, the group with dependents was more likely to be female (p < 0.01) and married or living with a partner (p < 0.01).

In Study 3, 29 of 126 participants were categorized as caregivers, including 19 who reported that they were parents of dependent children. Six reported being caregivers of adults, and four reported being both parents of dependent children and caregivers of adults (i.e., a total of 23 respondents were parents of dependent children). There were no statistically significant between-group differences in age, gender, employment status, or education level (Table 2). There were significant differences between caregivers and non-caregivers in ethnicity and marital status. Compared to non-caregivers, the caregiver group was more likely to be non-white (p = 0.02) and separated (p = 0.02).

Gender frequencies within each caregiver status group followed a different pattern in Study 1 than in Studies 2 and 3 (Table 2). Caregivers in Studies 2 and 3 were more likely to be female, whereas caregivers in Study 1 were more likely to be male. Because the gender frequencies in Study 1 were contrary to results from Studies 2 and 3, which had larger samples, this finding was confirmed by re-checking and re-tabulating gender and caregiver status from the original demographic questionnaires. This verification process confirmed that these gender statistics are reported correctly.

### Willingness to trade time or gamble

If the respondent is unwilling to trade any time in the TTO task or unwilling to gamble in the SG task, the resulting utility will be at the ceiling, which was 0.95 in the current analysis. Compared to non-caregivers, caregivers had fewer health state utilities less than 0.95 and more responses at the ceiling, indicating less willingness to trade time or gamble. This pattern was consistent across all three studies and across almost all health states. For all health states in Studies 1 and 2 (Tables 3 and 4), a smaller percentage of caregivers had utilities less than 0.95 compared with non-caregivers. In Study 3 (Table 4), a smaller percentage of caregivers had scores less than 0.95 for 14 of 16 health states (the exceptions were health states F and H). For example, in Study 1, while only 23.3% of caregivers were willing to trade time to avoid health state A, 46.0% of non-caregivers were willing to trade time. Chi-square analyses found that these differences between groups were statistically significant for two health states in Study 1 and two health states in Study 3. In sum, these findings indicate a greater ceiling effect for caregivers than for non-caregivers across all three samples.

**Table 3:** **Frequency and percentage of respondents with values less than 0.95**^**1 **^**(Study 1)**

**Health states**	**Caregivers**	**Non-caregivers**	**p-value**^**2**^
	**(N = 30)**	**(N = 50)**	
**Time trade-off**			
HS A: Mild Osteoarthritis	7 (23.3%)	23 (46.0%)	0.04
HS B: Moderate Osteoarthritis	12 (40.0%)	31 (62.0%)	0.06
HS C: Severe Osteoarthritis	21 (70.0%)	46 (92.0%)	< 0.01
HS D: Moderate Osteoarthritis + AE	14 (46.7%)	34 (68.0%)	0.06
**Standard gamble**			
HS A: Mild Osteoarthritis	5 (17.2%)	9 (18.4%)	0.90
HS B: Moderate Osteoarthritis	9 (31.0%)	16 (32.7%)	0.88
HS C: Severe Osteoarthritis	20 (69.0%)	40 (81.6%)	0.20
HS D: Moderate Osteoarthritis + AE	11 (37.9%)	20 (40.8%)	0.80

^1^Values less than 0.95 indicate willingness to trade in time trade-off choices and willingness to gamble in standard gamble choices. For the descriptive analyses in this table, ratings worse than dead were categorized as less than 0.95.

^2^Based on chi-square analyses comparing caregivers and non-caregivers.

HS = Health state.

AE = Adverse event.

**Table 4:** **Frequency and percentage of respondents with TTO utility values less than 0.95**^**1 **^**(Studies 2 and 3)**

**Health states**	**Caregivers**	**Non-caregivers**	**p-value**^**2**^
**Study 2 (Caregivers, n = 47, Non-caregivers, n = 111)**			
A. Adult ADHD, receiving treatment, responder	24 (51.1%)	65 (58.6%)	0.39
B. Adult ADHD, receiving treatment, non-responder	33 (70.2%)	86 (77.5%)	0.33
C. Adult ADHD, untreated	34 (72.3%)	88 (79.3%)	0.34
**Study 3 (Caregivers, n = 29, Non-caregivers, n = 97)**			
A. Basic HS (stable schizophrenia, no adverse events)	20 (69.0%)	73 (75.3%)	0.50
B. Basic HS + metabolic syndrome	22 (75.9%)	86 (88.7%)	0.08
C. Basic HS + weight gain	23 (79.3%)	78 (80.4%)	0.90
D. Basic HS + diabetes	21 (72.4%)	83 (85.6%)	0.10
E. Basic HS + hyperlipidemia	21 (72.4%)	83 (85.6%)	0.10
F. Basic HS + male sexual dysfunction	10 (83.3%)	39 (81.3%)	0.87
G. Basic HS + female sexual dysfunction	13 (76.5%)	41 (83.7%)	0.51
H. Basic HS + male increased prolactin levels	10 (83.3%)	40 (83.3%)	1.00
I. Basic HS + female increased prolactin levels	12 (70.6%)	43 (87.8%)	0.10
J. Basic HS + akathisia	22 (75.9%)	84 (86.6%)	0.17
K. Basic HS + tardive dyskinesia	24 (82.8%)	90 (92.8%)	0.11
L. Basic HS + other extrapyramidal symptoms	23 (79.3%)	91 (93.8%)	0.02
M. Basic HS + insomnia	20 (69.0%)	83 (85.6%)	0.04
N. Basic HS + somnolence	21 (72.4%)	83 (85.6%)	0.10
O. Basic HS + nausea	20 (69.0%)	80 (82.5%)	0.11
P. Basic HS + vomiting	21 (72.4%)	81 (83.5%)	0.18

^1^Values less than 0.95 indicate willingness to trade in time trade-off choices.

^2^Based on chi-square analyses comparing caregivers and non-caregivers.

HS = Health state.

ADHD = Attention deficit/hyperactivity disorder.

TTO = Time trade-off.

### Utilities

For every health state in all three samples, caregivers had higher utilities than non-caregivers. The difference in utility scores between caregivers and non-caregivers ranged from 0.07 to 0.16 for TTO utilities and 0.03 to 0.17 for SG utilities in Study 1 (Table 5). The between-group difference in TTO utilities ranged from 0.06 to 0.10 in Study 2, and from 0.11 to 0.22 in Study 3 (Table 6). In seven of the eight pairwise comparisons for Study 1, as well as all comparisons for Studies 2 and 3, the mean utility difference between caregivers and non-caregivers was sufficiently large to be considered clinically important (i.e., utility difference ≥ 0.05 [23]). The differences between caregivers and non-caregivers were statistically significant (p < 0.05) for some health states, including health states A and C in the TTO task in Study 1 and health state C in the SG task in Study 1 (Table 5). In Study 2, the between-group difference in TTO utilities was statistically significant for all three health states (Table 6). In Study 3, the between-group difference was statistically significant for health states J and P (Table 6).

**Table 5:** Health state utilities* (Study 1)

**Health states**	**Caregivers (N = 30)**			**Non-caregivers (N = 50)**			**Difference**	**t statistic**	**p-value**
	**n**^**†**^	**Mean (SD)**	**Min-Max**	**n**^**†**^	**Mean (SD)**	**Min-Max**			
**Time trade-off**									
HS A: Mild Osteoarthritis	30	0.92 (0.06)	0.75-0.95	50	0.85 (0.13)	0.45-0.95	0.07 (0.11)	3.2	<0.01
HS B: Moderate Osteoarthritis	30	0.84 (0.19)	0.05-0.95	49	0.76 (0.23)	0.00-0.95	0.08 (0.22)	1.6	0.11
HS C: Severe Osteoarthritis	24	0.69 (0.28)	0.15-0.95	42	0.53 (0.29)	0.05-0.95	0.16 (0.28)	2.3	0.03
HS D: Moderate Osteoarthritis + AE	30	0.82 (0.21)	0.05-0.95	48	0.72 (0.26)	0.00-0.95	0.10 (0.25)	1.8	0.07
**Standard gamble**									
HS A: Mild Osteoarthritis	29	0.93 (0.05)	0.75-0.95	49	0.90 (0.12)	0.45-0.95	0.03 (0.10)	1.4	0.16
HS B: Moderate Osteoarthritis	29	0.89 (0.10)	0.60-0.95	48	0.84 (0.20)	0.25-0.95	0.05 (0.17)	1.5	0.13
HS C: Severe Osteoarthritis	24	0.79 (0.19)	0.35-0.95	40	0.61 (0.33)	0.05-0.95	0.17 (0.28)	2.7	<0.01
HS D: Moderate Osteoarthritis + AE	29	0.88 (0.11)	0.60-0.95	47	0.81 (0.23)	0.15-0.95	0.07 (0.20)	1.7	0.09

^*^Based on t-tests comparing means of the two groups. Utility ratings worse than dead are not included in this table. In Study 1, a precise utility value was not obtained when a respondent considered a health state to be worse than dead (i.e., utility < 0). Therefore, participants who considered a health state to be worse than dead have missing data for that health state in this table.

^†^In Study 1, sample size varied for each individual health state because a specific utility value was not derived if a respondent believed that health state was worse than being dead.

HS = Health state.

AE = Adverse event.

**Table 6:** Time trade-off utilities* (Studies 2 and 3)

**Health states**	**Caregivers (N = 47)**			**Non-caregivers (N = 111)**			**Difference**	**t statistic**	**p-value**
	**n**	**Mean (SD)**	**Min-Max**	**n**	**Mean (SD)**	**Min-Max**			
**Study 2**									
A. Adult ADHD, receiving treatment, responder	47	0.87 (0.11)	0.45-0.95	111	0.80 (0.19)	−0.05-0.95	0.06 (0.17)	2.7	<0.01
B. Adult ADHD, receiving treatment, non-responder	47	0.75 (0.21)	0.05-0.95	111	0.65 (0.30)	−0.75-0.95	0.10 (0.28)	2.5	0.02
C. Adult ADHD, untreated	47	0.73 (0.23)	0.05-0.95	111	0.64 (0.30)	−0.65-0.95	0.10 (0.28)	2.2	0.03
**Study 3**									
A. Basic HS (stable schizophrenia, no adverse events)	29	0.68 (0.35)	−0.55-0.95	97	0.56 (0.45)	−0.75-0.95	0.11 (0.43)	1.3	0.21
B. Basic HS + metabolic syndrome	29	0.62 (0.35)	−0.55-0.95	97	0.44 (0.49)	−0.85-0.95	0.18 (0.46)	1.9	0.06
C. Basic HS + weight gain	29	0.64 (0.36)	−0.55-0.95	97	0.51 (0.47)	−0.85-0.95	0.12 (0.45)	1.3	0.19
D. Basic HS + diabetes	29	0.58 (0.39)	−0.55-0.95	97	0.43 (0.50)	−0.95-0.95	0.15 (0.48)	1.5	0.13
E. Basic HS + hyperlipidemia	29	0.61 (0.38)	−0.55-0.95	97	0.47 (0.47)	−0.85-0.95	0.13 (0.45)	1.4	0.16
F. Basic HS + male sexual dysfunction^**†**^	12	0.57 (0.29)	0.05-0.95	48	0.47 (0.45)	−0.85-0.95	0.11 (0.43)	0.8	0.44
G. Basic HS + female sexual dysfunction^**†**^	17	0.61 (0.43)	−0.55-0.95	49	0.45 (0.52)	−0.75-0.95	0.16 (0.50)	1.2	0.25
H. Basic HS + male increased prolactin levels^**†**^	12	0.62 (0.27)	0.05-0.95	48	0.44 (0.47)	−0.85-0.95	0.17 (0.44)	1.7	0.10
I. Basic HS + female increased prolactin levels^**†**^	17	0.63 (0.41)	−0.55-0.95	49	0.44 (0.50)	−0.75-0.95	0.19 (0.48)	1.4	0.17
J. Basic HS + akathisia	29	0.62 (0.35)	−0.55-0.95	97	0.43 (0.51)	−0.85-0.95	0.19 (0.48)	2.3	0.03
K. Basic HS + tardive dyskinesia	29	0.47 (0.49)	−0.85-0.95	97	0.25 (0.60)	−0.95-0.95	0.22 (0.58)	1.8	0.07
L. Basic HS + other extrapyramidal symptoms	29	0.41 (0.52)	−0.85-0.95	97	0.25 (0.57)	−0.95-0.95	0.16 (0.56)	1.4	0.17
M. Basic HS + insomnia	29	0.65 (0.38)	−0.55-0.95	97	0.49 (0.47)	−0.85-0.95	0.16 (0.45)	1.7	0.09
N. Basic HS + somnolence	29	0.64 (0.36)	−0.55-0.95	97	0.48 (0.47)	−0.85-0.95	0.16 (0.44)	1.7	0.10
O. Basic HS + nausea	29	0.62 (0.44)	−0.65-0.95	97	0.50 (0.48)	−0.95-0.95	0.11 (0.47)	1.1	0.26
P. Basic HS + vomiting	29	0.65 (0.36)	−0.55-0.95	97	0.45 (0.51)	−0.95-0.95	0.20 (0.48)	2.4	0.02

^*^Based on t-tests comparing means of the two groups.

^†^In Study 3, sample size was smaller for health states representing sexual dysfunction and increased prolactin because each participant rated only the gender-specific health state.

HS = Health state.

ADHD = Attention deficit/hyperactivity disorder.

Analyses were re-run as ANCOVA models, controlling for demographic variables that were statistically significant between groups (i.e., gender and marital status in Studies 1 and 2; ethnicity and marital status in Study 3). All differences between caregivers and non-caregivers that were statistically significant in the t-tests (Tables 5 and 6) were also statistically significant in these ANCOVA models. This suggests that between-group differences were due to caregiver status rather than other demographic differences. The ANCOVA models found one additional statistically significant difference between caregivers and non-caregivers (health state B in Study 3).

### Sensitivity analysis: parents

All analyses were re-run focusing on the 83 parents of dependent children (n = 25 in Study 1; n = 39 in Study 2; n = 19 in Study 3). These analyses followed the same patterns as those for the larger caregiver group. Compared with respondents who were not parents of dependent children, these parents were less likely to trade any time in TTO tasks for every health state rated across the three studies. This difference was statistically significant for five of the 23 health states (p < 0.05). In addition, these parents had lower TTO utility scores for every health state, with utility differences ranging from 0.06 to 0.14 in Study 1, 0.07 to 0.11 in Study 2, and 0.07 to 0.32 in Study 3. These utilities or differences were statistically significant for five of the 23 health states (p < 0.05). The SG results in Study 1 followed similar patterns, with lower utility scores and less willingness to gamble among these parents of dependent children than among the comparison group.

These analyses were also re-run focusing on the 173 participants who reported being parents, regardless of whether their children still depended on them for care (n = 33 in Study 1; n = 89 in Study 2; n = 51 in Study 3). These analyses followed the same patterns as those for the caregiver group and the group consisting of parents of dependent children. Compared with non-parents, these parents were less likely to trade any time in TTO tasks for every health state rated across the three studies. This difference was statistically significant for four of the 23 health states (p < 0.05). In addition, these parents had lower TTO utility scores than non-parents for every health state, with utility differences ranging from 0.05 to 0.19 in Study 1, 0.03 to 0.09 in Study 2, and 0.03 to 0.09 in Study 3. These utilities or differences were statistically significant for seven of the 23 health states (p < 0.05). The SG results in Study 1 followed similar patterns, with lower utility scores and less willingness to gamble among parents than among non-parents.

## Discussion

Taken together, results across three independent data sets for a wide range of health states provide strong support for the hypothesis that being a caregiver influences the way one responds in a TTO health state valuation task. Compared to respondents who were not caregivers, caregivers tended to be less willing to trade any time in the TTO tasks. Furthermore, when caregivers did trade, they tended to trade less time, resulting in consistently higher utility scores than non-caregivers. The difference between caregivers and non-caregivers in health state utility was often quite large, with between-group differences up to 0.22 on the standard utility scale anchored to 0 (dead) and 1 (full health). Utility differences of this magnitude are large enough to have a substantial impact on the outcome of a cost-utility model. Thus, caregiver status may be an important demographic variable to consider when collecting, interpreting, or using utility data. The current results also serve as a reminder that health state utilities are shaped not only by the health states themselves, but also by characteristics of the respondent valuing the health states.

SG data were not available in the data sets for Studies 2 or 3, but in Study 1, caregiver status appeared to have less influence on SG responses than on TTO responses. For example, caregiver status had a less pronounced impact on willingness to gamble in SG than on willingness to trade in TTO (Table 3). In addition, mean differences in utility scores between caregivers and non-caregivers were smaller for SG than TTO for three of the four health states in Study 1 (Table 5). Perhaps the SG task is less susceptible than TTO to the respondent’s current life situation because of the abstract nature of the task. In the TTO, participants may consider the direct impact of shorter lifespan on their dependents, whereas the probabilities involved in the SG task may not lead respondents to think of their own responsibilities as directly. Still, SG utilities had a smaller utility range for caregivers than for non-caregivers, the caregivers did have statistically significantly higher SG utilities for health state C, and between-group differences for health states B and D may be considered clinically important (Table 5[23]). In sum, while results of the TTO task appear to be more strongly influenced by caregiver status, SG responses may also be affected. Future research is needed to clarify the impact of caregiver status on SG responses.

Current findings add to results reported by van der Pol & Shiell [15] suggesting that people are less willing to trade time in the hypothetical TTO task if they know that others are directly dependent on them. Whereas the van der Pol & Shiell [15] study focused only on a sample of 30 parents of young children, the current study included both parents of dependent children and individuals who considered themselves to be caregivers of an adult. This broader group was used because respondents in previous utility assessment studies (conducted by authors of the current studies) have mentioned the impact of caring for an elderly or disabled relative. However, the great majority of caregivers in the current study cared for children rather than adults (i.e., 83 of 106 caregivers; 78.3%). As a sensitivity analysis, current analyses were re-run in two other subgroups: including parents of dependent children and parents of any child regardless of whether the child is still dependent. Results of these sensitivity analyses followed the same general pattern as results of the primary analysis. The parent subgroups were generally less willing to trade time (i.e., resulting in higher utility scores) than comparison groups. Future research, perhaps involving qualitative interviews asking participants to explain their TTO responses, is needed to explore the separate influence, if any, of parenting and other types of caregiving on utility scores. The current analysis may also be replicated in larger samples with various types of caregivers, including larger numbers of caregivers of adults.

Parenting and other types of caregiving may reduce willingness to trade due to increased altruism and a sense of responsibility for another individual’s well-being. In addition, even if a child is grown, being a parent may reduce willingness to trade because parents may want to live longer to experience specific milestones in their child’s life. For example, parents have reported that they wanted to live long enough to see their child’s wedding or graduation when responding in previous TTO studies conducted by the authors of the current study. Such extrinsic goals, which occur at a specific point in the future, have been proposed as one factor that could limit willingness to trade time in TTO or gamble in SG [24].

Current results also have implications for the conceptual foundations of utility assessments. The standard preference elicitation and quality-adjusted life year (QALY) models ask respondents to evaluate health states based on the effects on their own welfare. However, results reported in this paper and elsewhere in the literature suggest that respondents also consider the effects of their health on the welfare of others when valuing health states. Several authors have developed conceptual frameworks that broaden the utility model to take into account not only the preferences of respondents for their own health outcomes, but also their preferences for effects on other people such as spouses, children, and adult dependents. These frameworks are often referred to as a family or household utility function [25-27]. Current findings suggest that future research to develop and implement these family or household utility approaches could be useful, although this methodology could be complex, and the scores may not be entirely comparable to conventional utilities.

Study limitations related to sample selection should be acknowledged. During screening, efforts were made to avoid over-representing respondents from any particular age, ethnic/racial background, gender, or employment group. However, recruitment was not stratified or designed to be nationally representative in any of the three studies used for this secondary analysis. Furthermore, the recruitment strategy, requiring that all study participants respond to newspaper or online advertisements, could have introduced selection bias. The extent to which current results may differ from utilities derived from a nationally representative sample in the US, UK, or any other country is not known.

Another potential limitation is that a respondent’s personal experience with the condition described in health states could influence utility scores. In Studies 2 and 3, none of the participants were ever diagnosed with the condition described in the health states (ADHD and schizophrenia, respectively), although it is possible that they knew people who had received these diagnoses. It is not known whether participants in Study 1 had ever been diagnosed with the condition described in the health states (osteoarthritis). Personal knowledge of the conditions being rated could have an impact on utility scores, and it is not known whether this was a confounding factor in the current analysis.

It is also possible that aspects of the interview methodology could have introduced bias. For example, although health states were initially presented to participants in random order during ranking or VAS introductory tasks, health states were then presented in the TTO tasks in the order in which participants ranked them. At multiple points during the interviews and at the end of the interviews, participants were asked to review and compare all of their utility scores in order to minimize ordering effects and learning effects that could affect differences among health states. Furthermore, if there were any ordering or learning effects, any resulting biases would likely be the same for caregivers and non-caregivers, and consequently, this issue is unlikely to have an impact on the issues examined in the current secondary analysis.

## Conclusions

In sum, the current study indicates that parenting and potentially other types of caregiving appear to influence TTO utilities. Subsequent research may be conducted to replicate current findings in independent cohorts of parents with dependent children as well as among caregivers of adults. In future TTO studies, it may also be useful to assess parenting and adult caregiving status along with other demographic variables such as gender, age, and education. Samples with higher percentages of parents or caregivers may tend to yield higher utility scores. Thus, it may be helpful to know these respondent characteristics when interpreting data and comparing utilities across multiple studies.

## Abbreviations

TTO: Time trade-off; SG: Standard gamble; ADHD: Attention-deficit/hyperactivity disorder; NICE: National institute for health and care excellence; VAS: Visual analogue scale; QALY: Quality-adjusted life year; HS: Health states.

## Competing interests

Funding for this study was provided by Eli Lilly & Co. Three of the authors (Kristina Boye, Joseph Johnston, and Lee Bowman) are employees of Lilly, but their input into the conceptualization and interpretation of this study represented their own opinions, rather than those of the company. Louis Matza and Jessica Jordan are employees of Evidera, a company that received funding from Lilly for this research. David Feeny received funding from Lilly for time spent contributing to this research.

## Authors’ contributions

LM was the primary writer of this manuscript, and he directed the study including the study design, health state development (for studies 2 and 3), data collection, data analysis, and data interpretation. KB, JAJ, LB, and DF made substantial contributions to the study design, data interpretation, and resulting manuscript. JBJ co-wrote sections of the manuscript and assisted with study design. All authors provided input on multiple drafts of the manuscript and approval of the final draft. All authors read and approved the final manuscript.
